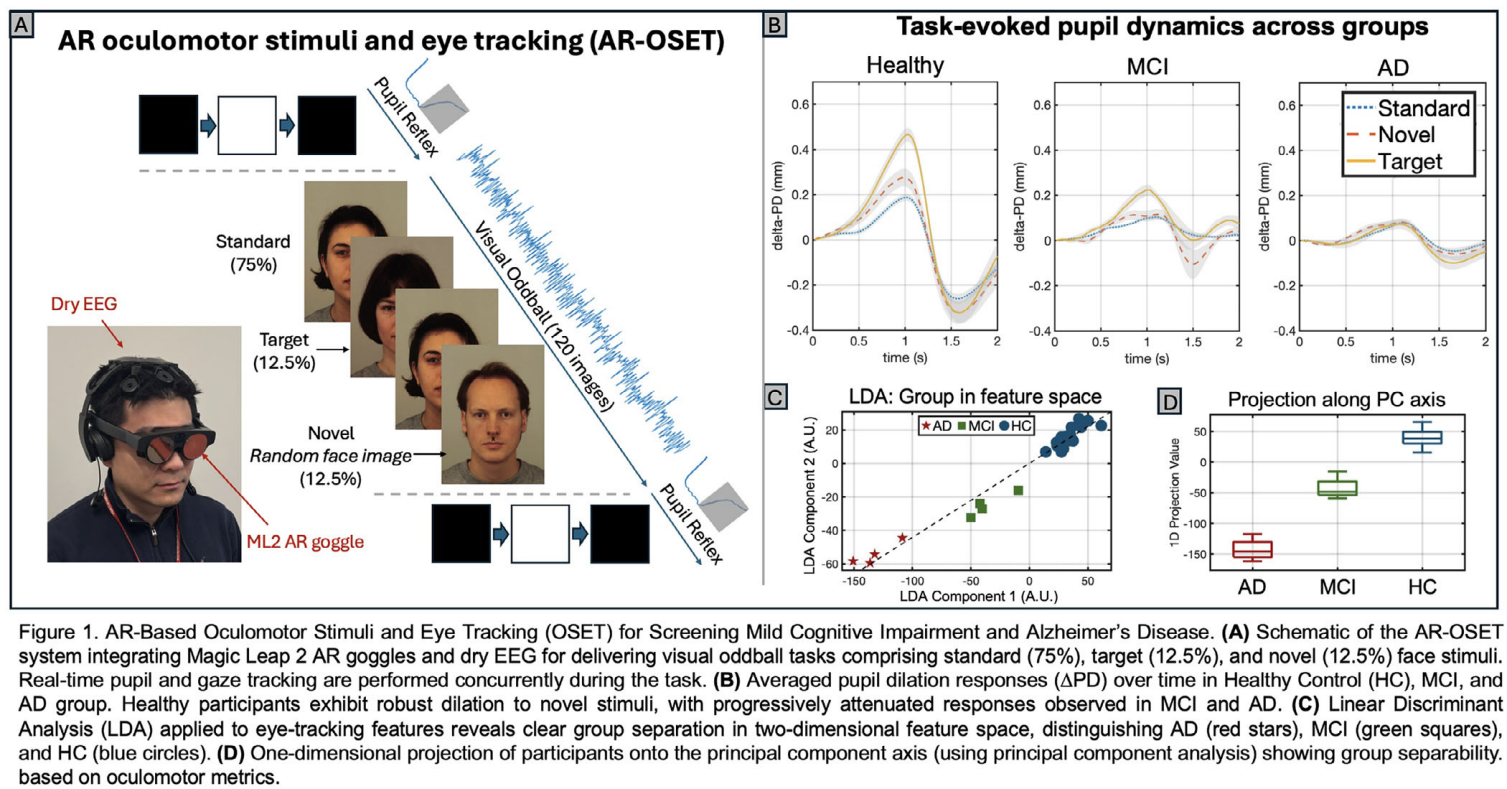# Wearable and Ambulatory Augmented Reality Oculomotor Stimuli and Eye‐Tracking for Early Detection and Monitoring of Alzheimer’s Disease

**DOI:** 10.1002/alz70861_108952

**Published:** 2025-12-23

**Authors:** Zipai Wang, Wanbin Tan, Gloria Chiang, Amirhossein Goldan

**Affiliations:** ^1^ Weill Cornell Medicine, New York, NY USA; ^2^ Weill Cornell Medicine, New York City, NY USA

## Abstract

**Background:**

Alzheimer’s disease (AD) is characterized by progressive cognitive decline associated with pathological changes originating in the locus coeruleus (LC), impacting oculomotor functions. Current diagnostic methods such as PET imaging and cerebrospinal fluid biomarkers are costly, invasive, and less accessible for routine screening. We propose a novel augmented reality (AR)‐based eye‐tracking system, the Oculomotor Stimulation and Eye Tracking (OSET) device, designed for non‐invasive, early detection and longitudinal monitoring of neurodegenerative disorders through immersive cognitive paradigms including anti‐saccade and oddball tasks.

**Method:**

The OSET device utilizes four infrared eye cameras with 60 Hz pupil tracking, with blink‐induced data loss compensated during preprocessing. Participants performed anti‐saccade and visual oddball task involving pseudo‐random presentation of emotionally neutral faces (75% standard, 12.5% target, 12.5% novel/randomly selected faces) sourced from the KDEF dataset. Each stimulus was presented for 800 ms with a fixed 2000 ms interstimulus interval, and participants responded to target faces using a handheld controller. A pupil reflex test using luminance transitions was conducted pre‐ and post‐task (Figure 1. A). Extracted features include peak delta pupil diameter (∆PD) in response to target and novel stimuli, standard error of the mean (SEM) for peak ∆PD values, pupil size dynamic range, total blink duration, and mean blink duration (Figure 1. B). Linear discriminant analysis (LDA) was used for dimensionality reduction and visualization of the group distributions.

The pilot cohort included two patients with AD, two with mild cognitive impairment (MCI), and 15 healthy controls, recruited at the Brain Health Imaging Institute (BHII) at Weill Cornell Medicine. Clinical diagnoses were established using the Clinical Dementia Rating (CDR) scale and tau‐PET imaging with Braak staging. Tau‐PET was served as the ground truth for validation. While the current sample size is limited, participant recruitment is ongoing to support broader validation.

**Result:**

Distinct oculomotor response patterns were observed across groups. The AR eye‐tracking system can differentiate among healthy controls, MCI, and AD patient based on oculomotor response patterns (Figure 1. C&D).

**Conclusion:**

The immersive, ambulatory, and cost‐effective nature of this system allows large‐scale longitudinal studies and frequent at‐home assessments, improving accessibility and patient compliance.